# The Effect of Repetitive Transcranial Magnetic Stimulation of Cerebellar Swallowing Cortex on Brain Neural Activities: A Resting-State fMRI Study

**DOI:** 10.3389/fnhum.2022.802996

**Published:** 2022-04-27

**Authors:** Linghui Dong, Wenshuai Ma, Qiang Wang, Xiaona Pan, Yuyang Wang, Chao Han, Pingping Meng

**Affiliations:** ^1^Department of Rehabilitation Medicine, Affiliated Hospital of Qingdao University, Qingdao, China; ^2^Department of Radiology, Affiliated Hospital of Qingdao University, Qingdao, China

**Keywords:** swallowing, repetitive transcranial magnetic stimulation (rTMS), motor evoked potentials (MEP), cerebellum, resting-state functional magnetic resonance imaging (rs-fMRI)

## Abstract

**Objective:**

The effects and possible mechanisms of cerebellar high-frequency repetitive transcranial magnetic stimulation (rTMS) on swallowing-related neural networks were studied using resting-state functional magnetic resonance imaging (rs-fMRI).

**Method:**

A total of 23 healthy volunteers were recruited, and 19 healthy volunteers were finally included for the statistical analysis. Before stimulation, the cerebellar hemisphere dominant for swallowing was determined by the single-pulse TMS. The cerebellar representation of the suprahyoid muscles of this hemisphere was selected as the target for stimulation with 10 Hz rTMS, 100% resting motor threshold (rMT), and 250 pulses, with every 1 s of stimulation followed by an interval of 9 s. The motor evoked potential (MEP) amplitude of the suprahyoid muscles in the bilateral cerebral cortex was measured before and after stimulation to evaluate the cortical excitability. Forty-eight hours after elution, rTMS was reapplied on the dominant cerebellar representation of the suprahyoid muscles with the same stimulation parameters. Rs-fMRI was performed before and after stimulation to observe the changes in amplitude of low-frequency fluctuation (ALFF) and regional homology (ReHo) at 0.01–0.08 Hz, 0.01–0.027 Hz, and 0.027–0.073 Hz.

**Results:**

After cerebellar high-frequency rTMS, MEP recorded from swallowing-related bilateral cerebral cortex was increased. The results of rs-fMRI showed that at 0.01–0.08 Hz, ALFF was increased at the pons, right cerebellum, and medulla and decreased at the left temporal lobe, and ReHo was decreased at the left insular lobe, right temporal lobe, and corpus callosum. At 0.01–0.027 Hz, ALFF was decreased at the left temporal lobe, and ReHo was decreased at the right temporal lobe, left putamen, and left supplementary motor area.

**Conclusion:**

Repetitive transcranial magnetic stimulation of the swallowing cortex in the dominant cerebellar hemisphere increased the bilateral cerebral swallowing cortex excitability and enhanced pontine, bulbar, and cerebellar spontaneous neural activity, suggesting that unilateral high-frequency stimulation of the cerebellum can excite both brainstem and cortical swallowing centers. These findings all provide favorable support for the application of cerebellar rTMS in the clinical practice.

## Introduction

Dysphagia is a common complication after stroke, and it has been documented that more than half of stroke patients have swallowing problems (Singh and Hamdy, 2006; Geeganage et al., 2012). Dysphagia may lead to various complications such as malnutrition, dehydration, pneumonia, prolonged hospitalization cycle, and even death (Bonilha et al., 2014; Cohen et al., 2016). The traditional rehabilitation methods for post-stroke dysphagia (PSD) include tongue muscle training, levator laryngeal muscle training, sensory stimulation training, dietary modification, and others, but these methods have poor efficacy (Martino and Mcculloch, 2016; Guillén-Solà et al., 2017). In order to accelerate the recovery speed and recovery rate of PSD patients, researchers are constantly exploring new treatment methods (Fisicaro et al., 2019).

Repetitive transcranial magnetic stimulation (rTMS) is a noninvasive brain stimulation technique, which can increase cortical excitability of the target area at high frequency (>1 Hz) and decrease it at low frequency (≤ 1 Hz) (Iglesias, 2020). Several studies have shown that recovery of swallowing function in PSD is associated with regulated excitability of the swallowing-related cortex and that rTMS treatment has a positive effect in patients with PSD (Khedr et al., 2009; Khedr and Abo-Elfetoh, 2010; Kim et al., 2011; Du et al., 2016; Cheng et al., 2017; Zhang et al., 2019). At present, the targets of rTMS treatment for PSD patients are mainly located in the cerebral cortex, but some patients have problems such as suboptimal efficacy and limited application, which promote clinicians to constantly search for new therapeutic targets.

As cerebellar function continues to be explored, multiple studies on healthy volunteers have shown that cerebellar rTMS may have a positive effect on swallowing function (Jayasekeran et al., 2011; Vasant et al., 2015; Sasegbon et al., 2019, 2020). This implies that the cerebellum has the potential to be a novel therapeutic target for PSD. Jayasekeran first demonstrated in 2011 that motor evoked potentials (MEPs) similar to those of pharyngeal constrictors can be generated in healthy humans by cerebellar single TMS. These findings suggested that cerebellar stimulation could induce swallowing movements in humans (Jayasekeran et al., 2011). Subsequently, sustained, high-frequency (5, 10, and 20 Hz) rTMS of unilateral hemispherical cerebellum showed significantly increased MEP amplitude at the swallowing-related cortex of both cerebral hemispheres, with maximum effect maintained for at least 30 min at 250 pulses (Vasant et al., 2015).

In order to verify whether the excitatory cortical effect produced by cerebellar rTMS can improve PSD, Sasegbon (Sasegbon et al., 2019, 2020) simulated post-stroke swallowing disorder in healthy volunteers using a total of 600 low-frequency pulses for 10-min inhibitory stimulation in the cortical region representative of pharyngeal constrictor muscle. Following this, rTMS (10 Hz, 250 pulses) was performed on the cerebellum, and results indicated that unilateral or bilateral stimulation could completely reverse simulated dysphagia and improve the excitability of the cortex related to bilateral cerebral swallowing, with bilateral stimulation more effective than unilateral stimulation. However, the above studies using electrophysiology show changes in the excitability of swallowing-related cortex but not the mechanism by which cerebellar rTMS affects this cortical region, or whether cerebellar stimulation affects other swallowing-related networks. Before cerebellar rTMS can be applied clinically, its effects on brain function need to be explored.

Recently, resting-state functional magnetic resonance imaging (rs-fMRI) has played an important role in studying brain functional activity as a safe, noninvasive technique, which reflects neural activity *via* blood oxygen level-dependent (BOLD) signals (Mosier et al., 1999; Mosier and Bereznaya, 2001; Suzuki et al., 2003; Zhang et al., 2020; Ma et al., 2021). Amplitude of low-frequency fluctuations (ALFF) and regional homogeneity (ReHo) are two classic local indices of rs-fMRI, which are widely used due to good stability (Qiu et al., 2019; Wu et al., 2020). ALFF has been calculated as the mean amplitude of the BOLD signal deviating from baseline over a short period of time, reflecting the strength of the spontaneous neural activity in voxels (Zang et al., 2007). ReHo can indirectly reflect the synchrony of neural activity in local brain regions by calculating the time series consistency between the response at each voxel and its neighbors (Zang et al., 2004). Increased ReHo indicates that neural activity in local brain regions is synchronized. Reduced ReHo indicates a consistent reduction in neural activity.

Most of the current research on rs-fMRI focuses on low-frequency oscillatory signals in the classical frequency band (0.01–0.08 Hz), which are considered most relevant to neural activity. However, it has also been found that sensitivity to oscillatory signals in different frequency bands varies with brain regions and that sensitivity to wider frequency bands is reduced in some brain regions, leading to reduced detection of brain activity in those regions. Therefore, ALFF and ReHo are assessed in response to several different frequency bands, such as the slow-4 band (0.027–0.073 Hz) and the slow-5 band (0.01–0.027 Hz), for more targeted investigation of brain functional activity (Zuo et al., 2010).

In this study, we used the multifrequency ALFF and ReHo methods to observe regional changes in brain function after high-frequency rTMS of the cerebellum. The study aimed to explore the effects and mechanisms of cerebellar rTMS on swallowing-related neural networks and ultimately to facilitate its clinical application.

## Materials and Methods

### Participants

This study included 23 healthy volunteers recruited at the Department of Rehabilitation, the Affiliated Hospital of Qingdao University, from September 2020 to February 2021. The study was reviewed by the ethics committee of the Affiliated Hospital of Qingdao University (ethics approval number: qyfy wzll 26154), and all volunteers provided a signed declaration of informed consent for participation. All volunteers received unilateral (dominant side) cerebellar hemisphere 10 Hz, 250 pulse rTMS stimulation.

#### Inclusion Criteria

1). Age ≥ 18 years.
2). No history of diseases, such as Parkinson's disease or stroke, which may cause dysphagia.
3). No contraindications to the use of rTMS or to fMRI (history of seizures, intracorporeal implantation of pacemaker or drug pump, cranial metal implant, claustrophobia, and others).
4). Normal cognitive function and ability to cooperate with the study procedures.

#### Exclusion Criteria

1). Current use of drugs affecting the central nervous system.
2). Current pregnancy, late-stage malignancy, history of brain surgery, or central nervous system disease.
3). Mandibular skin breakdown, infection, and other effects of surface electrode sheet placement.
4). Combined heart, lung, liver, kidney, and other important organ diseases, and if the condition is critical.

### Experimental Procedure

One group of healthy volunteers was included. Single-pulse TMS was used to stimulate bilateral cerebellar hot-pot of suprahyoid muscles, observing and comparing the bilateral resting motor threshold (rMT) and MEP. The dominant cerebellar hemisphere was defined as that with lower rMT, or if rMT was symmetrical, higher MEP was observed in the hemisphere. The dominant cerebellar representation of suprahyoid muscles was stimulated with 10 Hz rTMS, 100% rMT, and 250 pulses, with every 1 s of stimulation followed by an interval of 9 s. Before and after stimulation, the MEP amplitude of bilateral cerebral representation of suprahyoid muscles was measured to evaluate the excitability of swallowing-related cerebral cortex.

**Figure 1:**
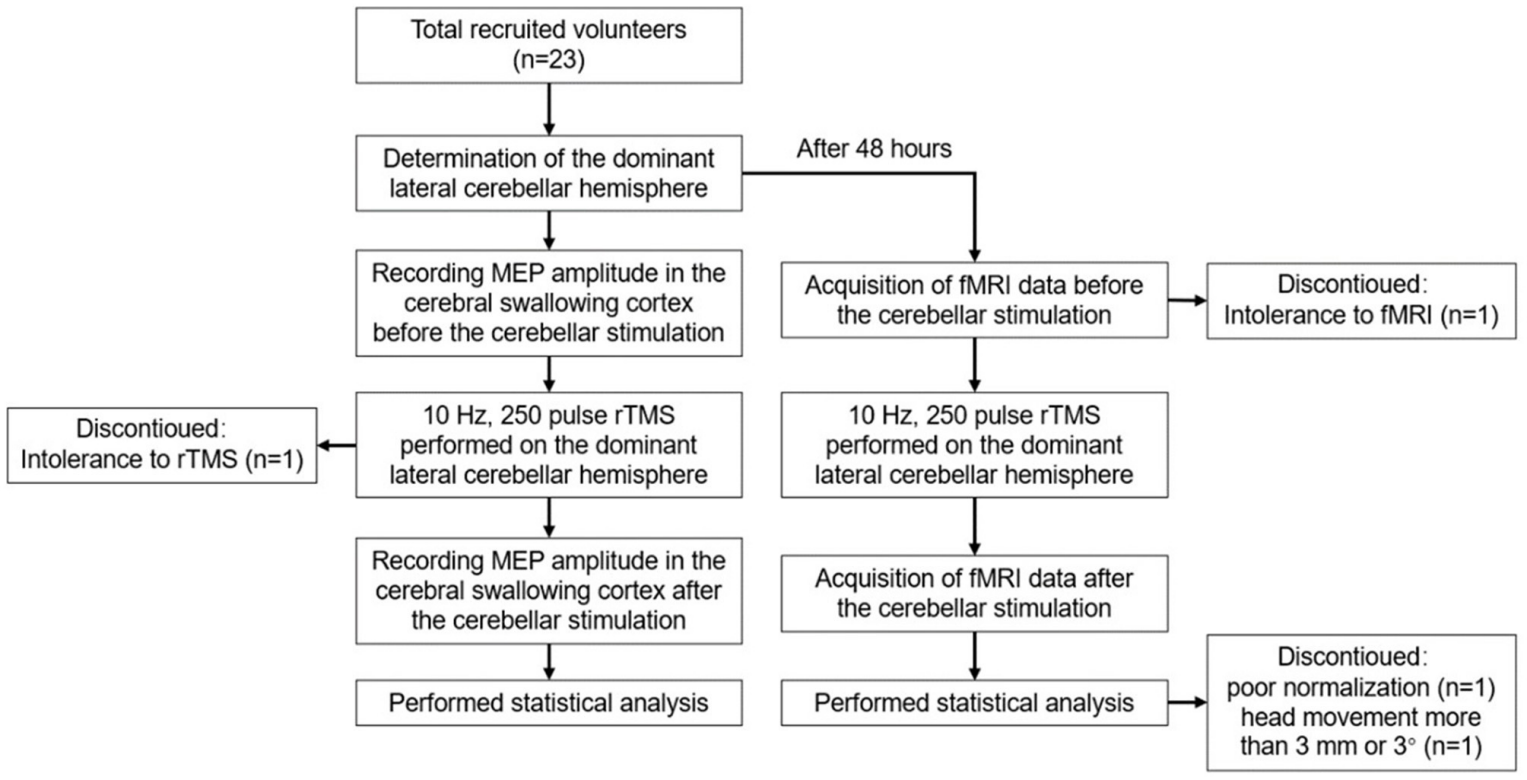
The study design and flowchart.

Forty-eight hours after elution, rTMS was repeated using the same parameters. Rs-fMRI was performed before and after stimulation to observe changes in ALFF and ReHo at 0.01–0.08 Hz, 0.01–0.027 Hz, and 0.027–0.073 Hz. Figure 1 shows the study design and flow chart.

### TMS and Electromyography

A circular coil (outer ring diameter: 70 mm) connected to CCY-IA TMS (Yiruide CCY-IA, Wuhan, China) was used, with a maximum stimulator output of 3.0 Tesla. Volunteers were seated, and alcohol was used to cleanse the neck skin and increase electrode conductivity. The circular coil was positioned at 45° tangential to the skull, and suprahyoid muscles electromyography was recorded *via* surface electrodes. The recording electrode was placed 2 cm left and right at the midpoint of the line connecting the mandible to the middle of the hyoid bone, and the reference electrode was affixed to the angle of the mandible. Coil movements were made in a region of 2–4 cm anterior and 4–6 cm lateral to the vertex of the skull, using 80% output, to obtain the maximal MEP at the location of suprahyoid muscles' motor representation of the cerebral cortex. Similarly, to find the cerebellar representation of suprahyoid muscles, the coil was moved around 1 cm lateral to and below the occipital external carina.

#### Resting MT

Resting MT was defined as the lowest TMS intensity that can evoke MEP amplitude greater than 50 μV in five out of ten trials, expressed as a percentage of the stimulator's maximum output intensity.

#### The MEP Amplitude

The MEP amplitude used for comparison was measured in the bilateral cerebral motor representation of the suprahyoid muscles, with 100% rMT as the stimulation intensity, measured for five times, and the average value was taken.

### Image Data Acquisition

A Signa HDX 3.0T (GE Healthcare, USA) nuclear magnetic resonance instrument was used to collect fMRI data. Volunteers were in the supine position with their head position fixed bilaterally using foam pads, eyes closed using an eye mask, and earplugs to protect hearing. Volunteers were instructed to relax, slow their breathing, and refrain from falling asleep during the scanning procedure. The fMRI scanning procedure began with a 3PL localizer scan, followed by an assessment calibration, and a subsequent blood signal scan. A total of 128 volumes were acquired using an echo-planar imaging sequence (30 axial slices, repetition time = 3,000 ms, echo time = 40 ms, flip angle = 90°, matrix = 128 × 128, in-plane resolution of 1.875 mm × 1.875 mm, thickness/gap = 5/0 mm). Subsequently, 3D T1-weighted anatomical images were acquired (248 sagittal slices, repetition time = 5.5 s, echo time =1.7 ms, matrix = 256 × 256, voxel size 1 mm × 1 mm × 1.2 mm).

### Image Data Preprocessing

Based on the Matlab 2018a software platform, preprocessing was performed using RESTplus v1.24. The image of the left dominant side was flipped to the right before statistical analysis, using a new version of RESTplus_v1.25.

#### Image Preprocessing

Image preprocessing was conducted using REST plus v1.24 as follows: (1) Data conversion from DICOM to Neuroimaging Informatics Technology Initiative format; (2) Removal of the first 10 time points; (3) Slice timing; (4) Image realignment; (5) Image normalization; (6) Image detrending; (7) Nuisance covariates regression: Friction 24, white matter signal, and cerebrospinal fluid signal; (8) ALFF and ReHo were calculated at three filtered bands: 0.01–0.08 (classical frequency band), 0.01 – 0.027 (slow-5), and 0.027–0.073 (slow-4); and (9) Data exclusion due to poor normalization or head movement more than 3 mm or 3°.

The global mean ALFF (mALFF) maps and mean fractional ALFF (mfALFF) maps at three bands were calculated for each subject using RESTplus v1.82 software in MATLAB prior to statistical analysis.

#### Smoothing

The mALFF and mean ReHo (mReHo) in the above frequency bands were smoothed using a 4 x 4 x 4 kernel in SPM software version 12.

#### Flip

Subjects turning to the right in response to the left side of the stimulus used the software package RESTplus v1.25.

### Statistical Analysis

SPSS version 22.0 was used to conduct paired samples *t*-tests comparing the MEP amplitude before and after stimulation.

Resting-state fMRI analysis was conducted using paired *t*-tests and FD_Power taut regression as a covariate in the Matlab 2018a software platform and the DPABI V5.1 software package. The resulting T-maps were Gaussian random field corrected, voxel *p* < 0.05, cluster *p* < 0.05, two-tailed test, default corner connected, default cluster size adopted, and finally output reported.

## Results

### Participants

Of a total of 23 healthy volunteers recruited for this study, two withdrew due to poor compliance or tolerance of the rTMS and fMRI procedures, and data from another two were excluded from preprocessing due to movement exceeding 3 mm or 3°, or poor registration. Therefore, data from 19 participants were included (thirteen female and six male participants, mean age 25.53 ± 4.29 years). In six out of the 19 participants, the dominant cerebellar hemisphere was on the right, while in others, it was on the left.

### MEP Amplitude Changes in Cerebral Swallowing Cortex

**Figure 2:**
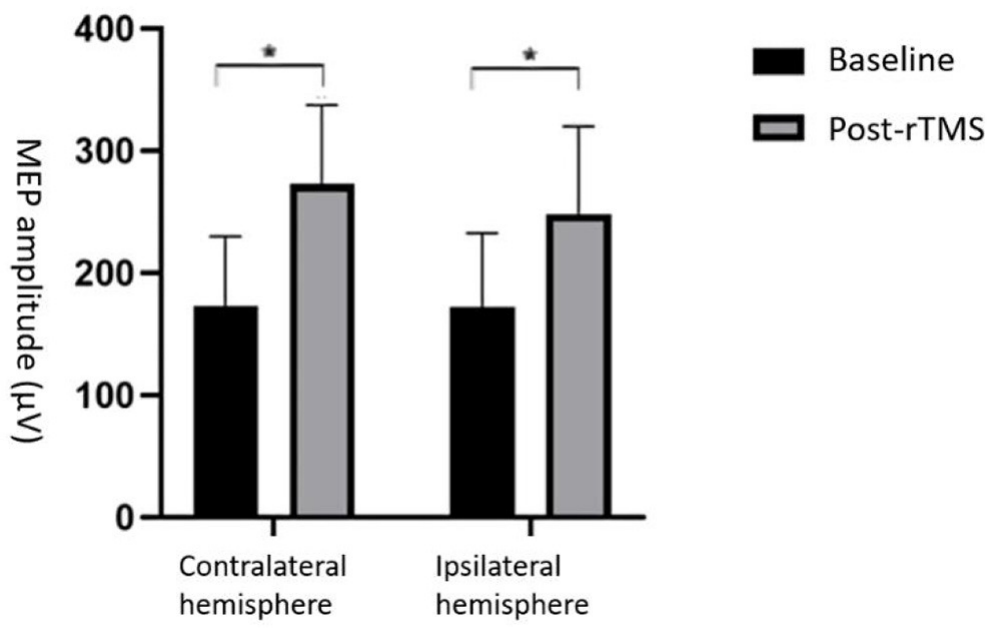
Comparison of MEP amplitude between rTMS and baseline. MEP amplitude increased in both cerebral hemispheres *p < 0.05.

We found that the MEP amplitude in the swallowing-related cortex was significantly elevated in both ipsilateral and contralateral hemispheres after stimulation (*p* < 0.05; Figure 2), indicating that high-frequency rTMS stimulation of the dominant lateral cerebellum can induce bilateral elevation of the swallowing cortex excitability.

### Rs-fMRI Analyses Under Different Frequency Bands

#### Resting-State fMRI Results Based on the ALFF Method

**Figure 3:**
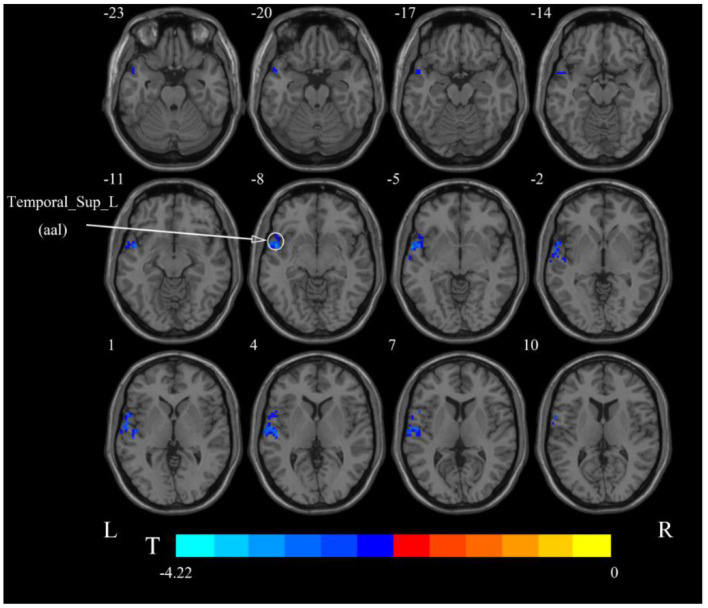
Statistical maps showing ALFF change pre- and post-rTMS in the classical frequency band. Warm colors showing ALFF increased and cool colors showing ALFF decreased after rTMS *p* < 0.05.

**Figure 4:**
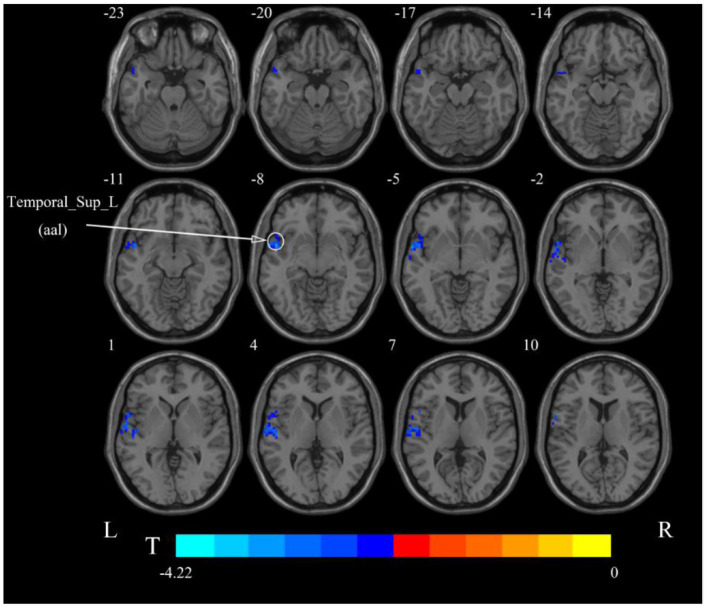
Statistical maps showing ALFF change pre- and post-rTMS in the slow-4 band. Cool colors showing ALFF decreased after rTMS *p* < 0.05.

**Figure 5:**
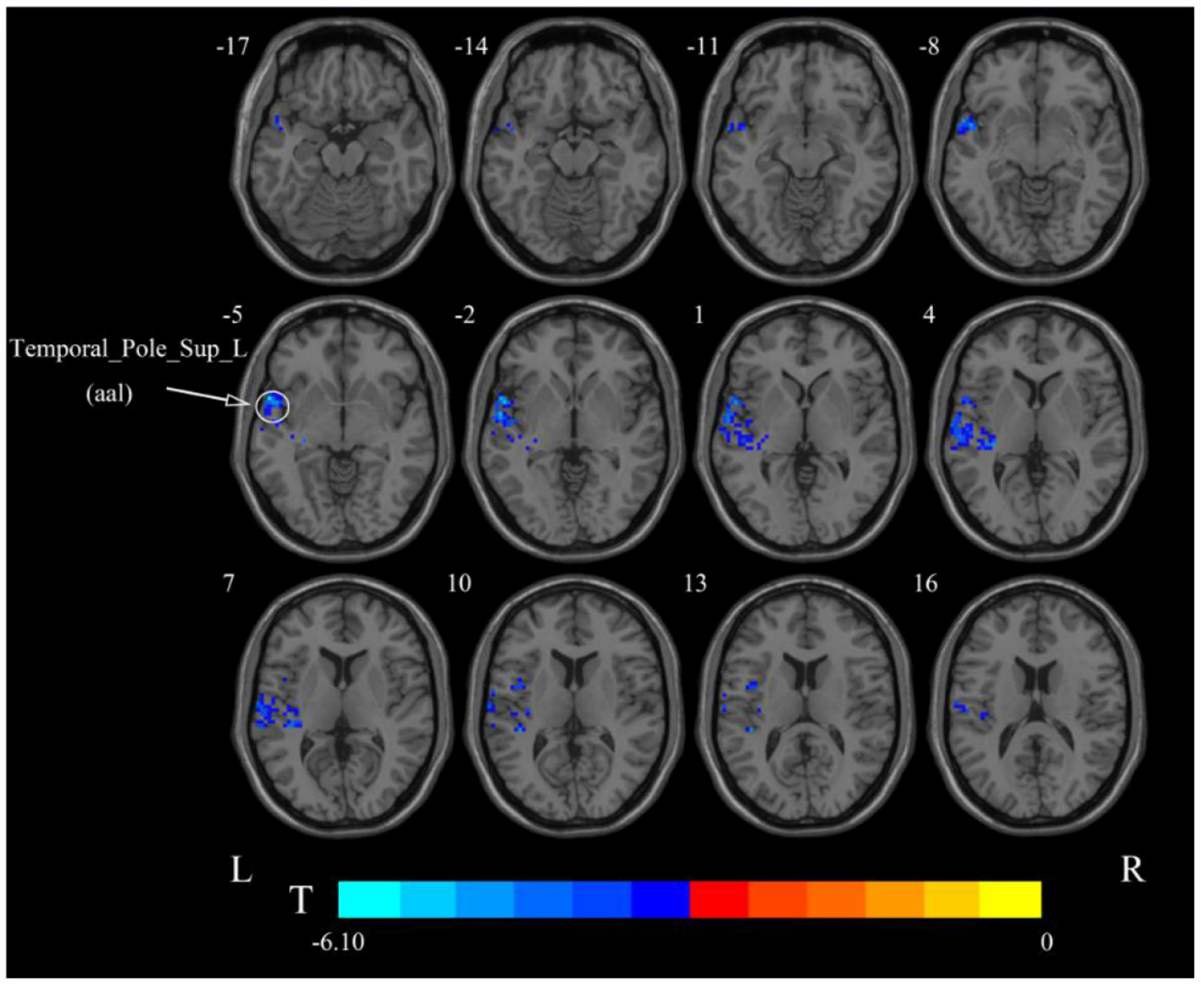
Statistical maps showing ALFF change pre- and post-rTMS in the slow-5 band. Cool colors showing ALFF decreased after rTMS *p* < 0.05.

After cerebellar stimulation, in the classical frequency band we found that ALFF was significantly elevated at the pons, right cerebellum, and medulla and significantly reduced at the left temporal lobe (*p* < 0.05; Figure 3 and Table 1), indicating that unilateral cerebellar stimulation can produce increased spontaneous neural activity in the cerebellum and brainstem and suppress activity at the contralateral temporal lobe. In addition, we found that ALFF at the left temporal lobe was also decreased in the slow-4 band and slow-5 band (*p* < 0.05; Figures 4, 5 and Table 1).

**Table 1:** Brain regions with alteration of ALFF after cerebellar rTMS.

**Brain region**	**Cluster size (voxel)**	**Coordinates (x, y, z)**	**Peak *t*-value**
**Classical frequency band**			
**(0.01–0.08 Hz)**			
Brainstem	149	12, −42, −39	3.972
Pons	67		
Cerebellum_9_R (aal)	21		
Medulla	19		
Temporal_Sup_L (aal)	260	−51, 3, −9	−4.6805
**Slow-4 band (0.027–0.073 Hz)**			
Temporal_Sup_L (aal)	175	−60, 6, −9	−4.2171
**Slow-5 band (0.01–0.027 Hz)**			
Temporal_Pole_Sup_L (aal)	292	−57, 9, 6	−6.1004

*aal, anatomical automatic labeling; L, left; R, right; T, statistical value of peak voxel showing ALFF changes pre- and post-rTMS (negative values: ALFF decreased after rTMS; positive values: ALFF increased after rTMS)*.

#### The Results of rs-fMRI Based on ReHo Method

**Figure 6:**
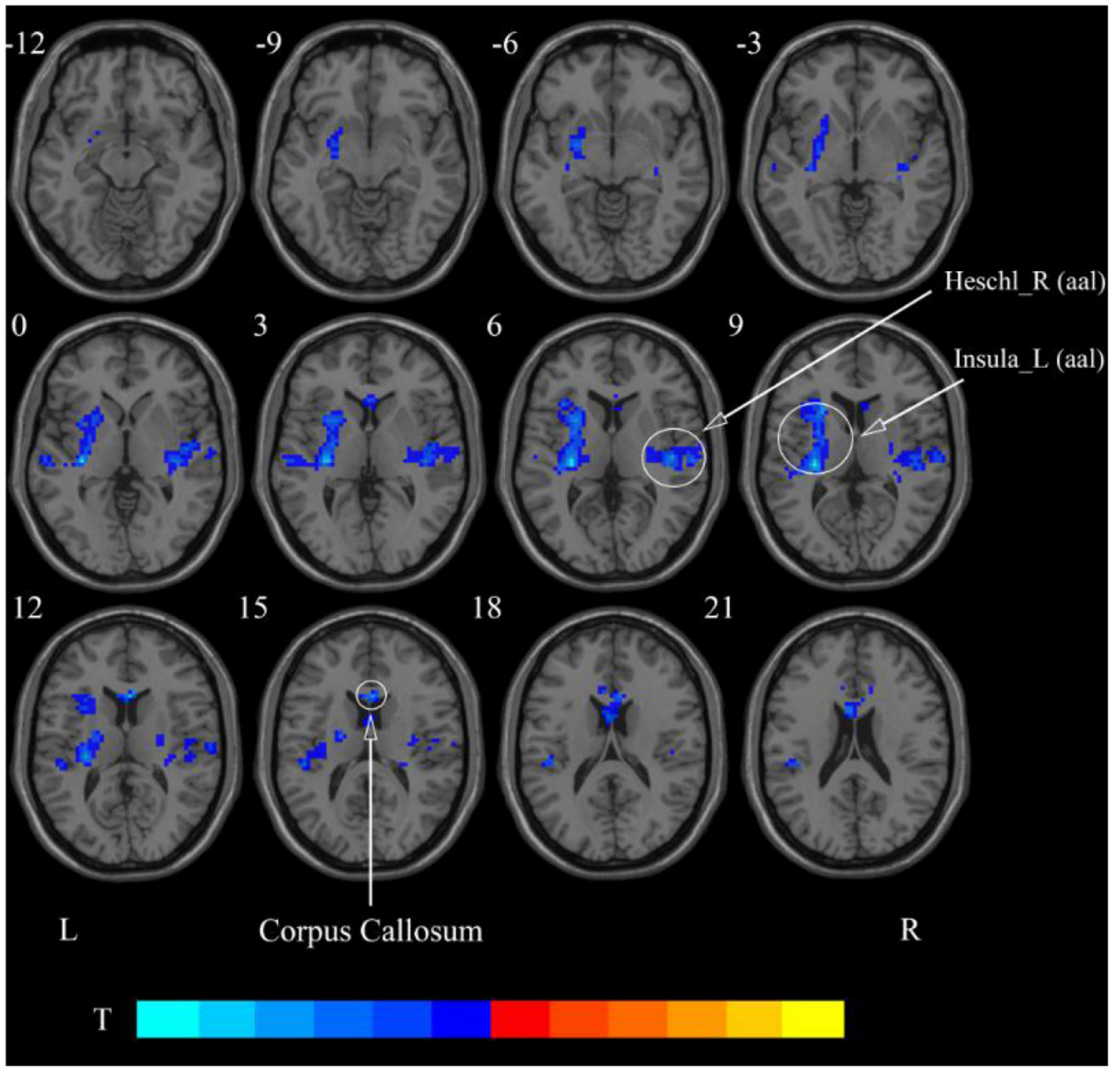
Statistical maps showing ReHo change pre- and post-rTMS in the classical frequency band. Cool colors showing ReHo decreased after rTMS *p* < 0.05.

**Figure 7:**
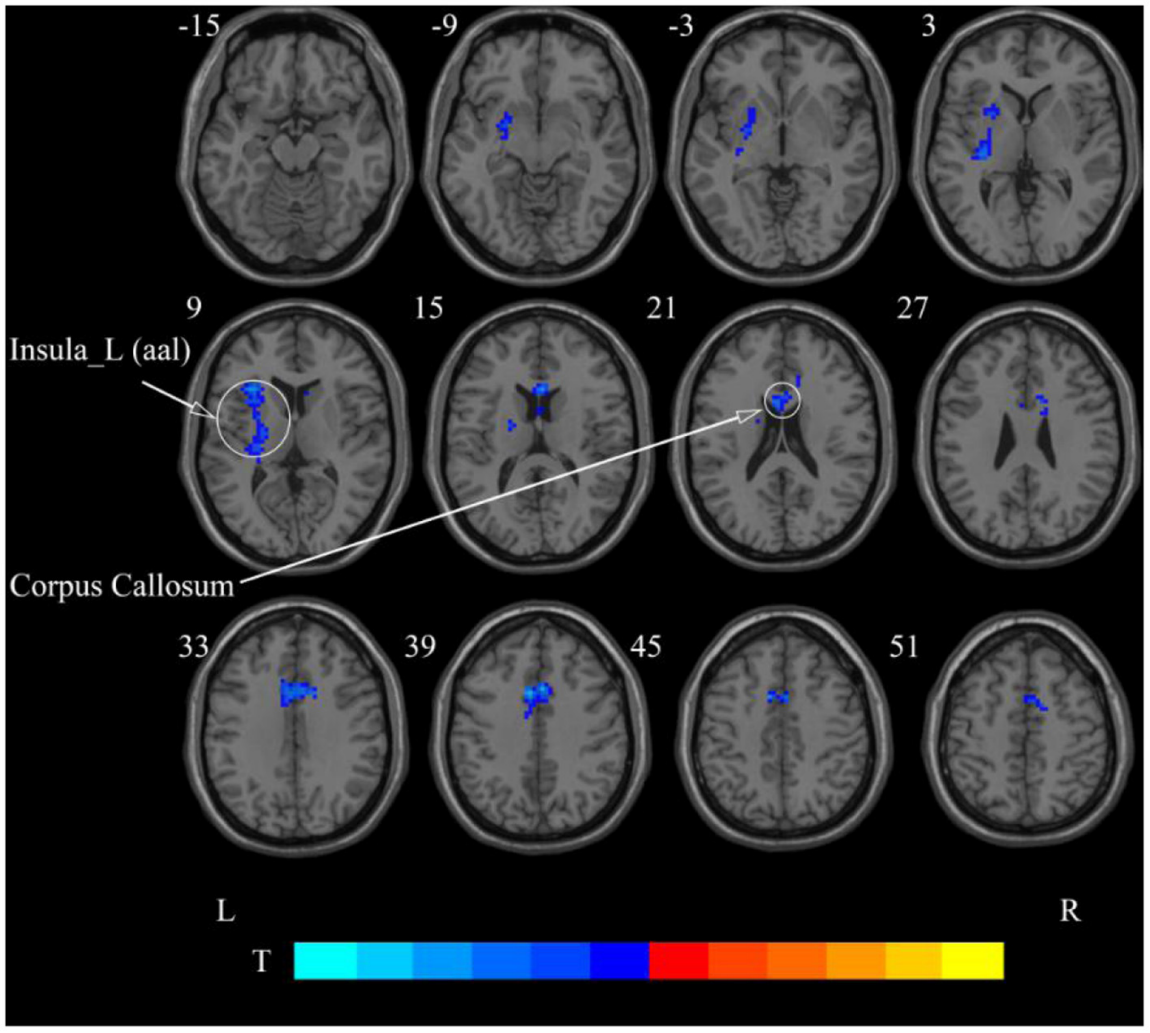
Statistical maps showing ReHo change pre- and post-rTMS in the slow-4 band. Cool colors showing ReHo decreased after rTMS *p* < 0.05.

**Figure 8:**
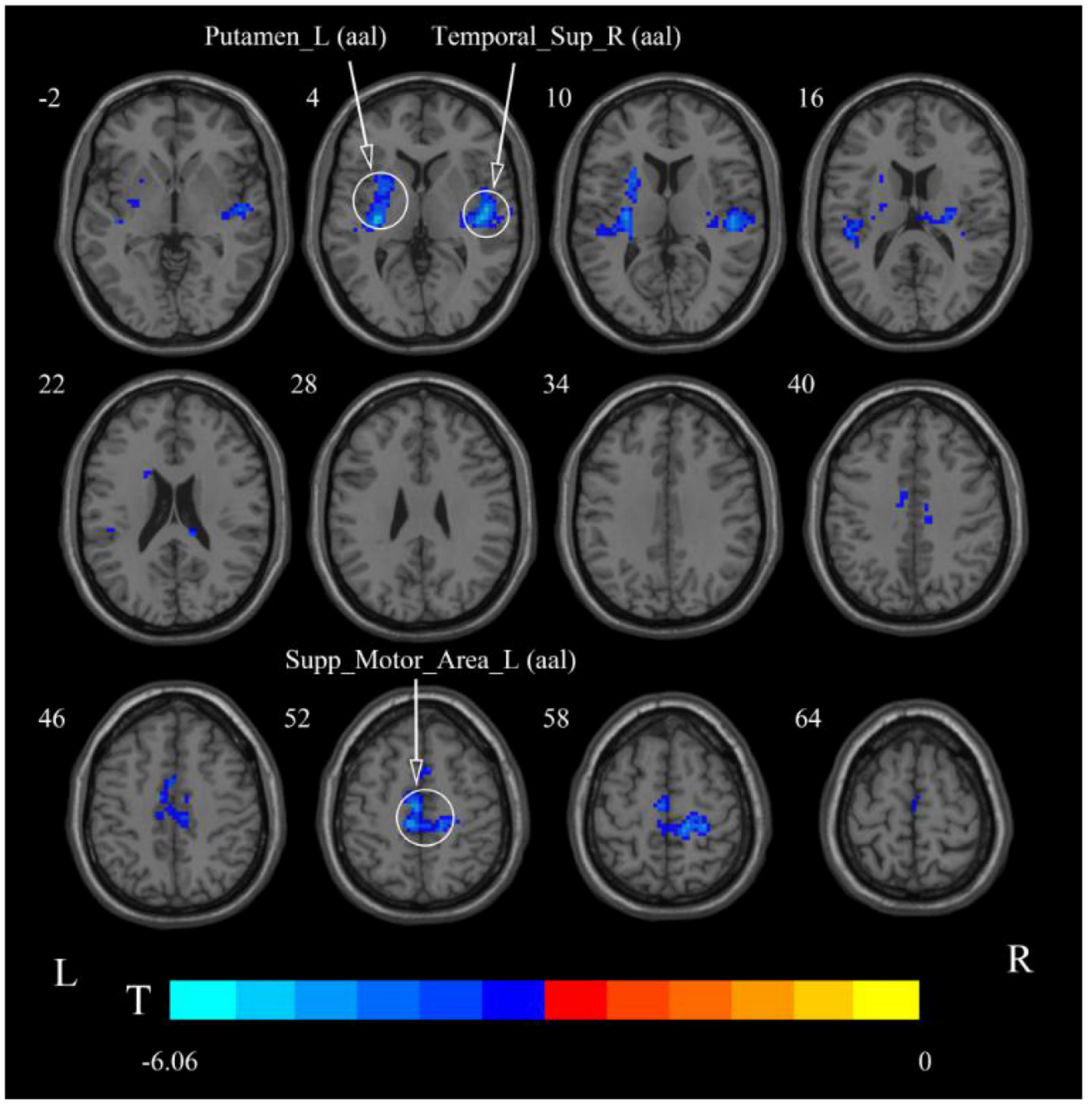
Statistical maps showing ReHo change pre- and post-rTMS in the slow-5 band. Cool colors showing ReHo decreased after rTMS *p* < 0.05.

After cerebellar stimulation, in the classical frequency band we found significantly decreased ReHo at the left insula, right temporal lobe, and corpus callosum (*p* < 0.05; Figure 6 and Table 2). In addition, a decrease in ReHo was also found at the left insula and corpus callosum in the slow-4 band and at the right temporal lobe, left putamen, and left motor accessory area in the slow-5 band (*p* < 0.05; Figures 7, 8 and Table 2).

**Table 2:** Brain regions with alteration of ReHo after cerebellar rTMS.

**Brain region**	**Cluster size (voxel)**	**Coordinates (x, y, z)**	**Peak *t*-value**
**Classical frequency band**			
**(0.01–0.08 Hz)**			
Insula_L (aal)	506	−30, −24, 9	−5.9848
Heschl_R (aal)	253	45, −18, 6	−4.0475
Corpus Callosum	109	3, 21, 12	−5.4896
**Slow-4 band (0.027–0.073 Hz)**			
Insula_L (aal)	274	−30, 21, 9	−4.4308
Corpus callosum	8		
**Slow-5 band (0.01–0.027 Hz)**			
Temporal_Sup_R (aal)	312	45, −18, 3	−4.8464
Putamen_L (aal)	354	−30, −18, 6	−5.1193
Supp_Motor_Area_L (aal)	367	−6, −15, 54	−6.0648

*aal, anatomical automatic labeling; L, left; R, right; T, statistical value of peak voxel showing ReHo changes pre- and post-rTMS (negative values: ReHo decreased after rTMS)*.

## Discussion

By examining neuroimaging and electrophysiological changes after rTMS, this study demonstrated that cerebellar rTMS may improve swallowing function. The electrophysiological findings are consistent with those of previous studies, showing that MEP amplitude at swallowing-related areas of bilateral cerebral hemispheres increased after unilateral cerebellar high-frequency stimulation (Vasant et al., 2015; Sasegbon et al., 2019, 2020). This means that high-frequency rTMS stimulation of the unilateral cerebellum can have a positive effect on the swallowing-related cortex of bilateral cerebral hemispheres. In addition to this, rs-fMRI results based on the ALFF method showed that neural activity in the pons and medulla was significantly enhanced after stimulation, providing the first validation that high-frequency stimulation of the cerebellar dominant hemisphere can produce excitatory brainstem effects. These findings provide supportive evidence for the application of cerebellar rTMS in the treatment of PSD.

### ALFF Results in Different Frequency Bands

In the classical frequency band, ALFF of posterior pons and medulla oblongata after cerebellar rTMS increased after stimulation, suggesting that brainstem spontaneous neural activity may be enhanced by high-frequency rTMS stimulation in the dominant cerebellar hemisphere. The generation of physiological swallowing activity requires oropharyngeal sensory stimulation afferent to the swallowing control center of the brainstem. This process is mainly mediated by the brainstem, whereas cortical higher-order centers act to initiate and regulate voluntary swallowing (Torii et al., 2012). The brainstem swallowing center, also called the central pattern generator (CPG), is located dorsolaterally in the medulla oblongata and is responsible for controlling and modulating the swallowing reflex. The CPG includes two bilaterally symmetrical regions, namely, the dorsal region comprising the nucleus tractus solitarius and its reticular formation; and the ventral region comprising the nucleus ambiguus and its reticular formation (Jean, 2001). Under physiological conditions, there is bilateral synergy between the two CPG regions, and their nerve fibers cross the midline in the brainstem to induce contraction of swallowing-related muscle groups bilaterally (Aydogdu et al., 2001). As a swallowing center, the brainstem is crucial in the swallowing process, and elevated ALFF values at the pons and medulla imply that high-frequency rTMS of the cerebellar dominant hemisphere may play a positive role on the efferent process of swallowing movements.

We also observed elevated ALFF in the cerebellum in the classical band. The cerebellum is a major site for the coordination of fine limb movements (Glickstein et al., 2009; Stoodley and Schmahmann, 2009). Several studies have previously shown that the cerebellum similarly plays an important role in swallowing movements. For example, fMRI research shows significant activation of the cerebellum during the oral phase of swallowing movements and coordinated orofacial and labial lingual movements (Onozuka et al., 2002). In addition, the accuracy of swallowing can be improved by cerebellar rTMS as measured using challenging swallow response tasks (Mistry et al., 2007; Sasegbon et al., 2019), and this finding may reflect the cerebellum increasing its role in modulating fine motor activity, thereby increasing movement accuracy.

In addition, we also found decreased ALFF in the contralateral temporal lobe after cerebellar rTMS, suggesting a possible inhibitory effect of cerebellar rTMS on the contralateral temporal lobe. Previous studies have shown that swallowing is governed by multiple parts of the cerebral cortex, such as the sensory/motor cortex, prefrontal areas, anterior cingulate, insula, parietal and temporal lobes (Hamdy et al., 1999; Martin et al., 2001; Suzuki et al., 2003; Babaei et al., 2012, 2013; Ushioda et al., 2012). The temporal lobe is thought to be involved in taste recognition during swallowing (Small et al., 1999). Since the swallowing action directly induced by cerebellar rTMS skips taste recognition, the temporary lobe suppression may be related to the negative feedback between cerebellum and temporal lobe. The cortical ALFF results showed an inhibitory effect of cerebellar rTMS on the contralateral temporal lobe in both the slow-4 and slow-5 frequency bands. However, unlike the classical band, no functional changes in the pons, medulla oblongata, or cerebellum were found in either frequency band. This suggests that the activity of these regions may be manifested in the entire classical frequency band rather than in some part of it.

### ReHo Results in Different Frequency Bands

In the classical frequency band, we observed that functional changes in the cerebral cortex were induced after rTMS stimulation in the cerebellum. The ReHo was decreased after stimulation in the left insula and right temporal lobe. The insula and temporal lobe function, similar to their involvement in swallowing, are jointly involved in taste recognition (Ertekin and Aydogdu, 2003). In addition to taste, the insula processes information such as food touch in the mouth and plays an important role in oral motility (Ushioda et al., 2012). We observed reduced ReHo in the temporal lobe, putamen, and motor supplementary area at slow-5, which may be more sensitive at slow-5. The supplementary motor area is involved in the planning of swallowing movements and has a role in coordinating bilateral movements (Welniarz et al., 2019; Sadler et al., 2021). The cerebral cortex plays a role in the initiation and regulation of voluntary swallowing (Suzuki et al., 2003). After stimulation of the cerebellum, the spontaneous neural activity of the stimulated lateral cerebellum and brainstem was enhanced, while multiple regions of the cerebral cortex were negatively affected. Swallowing movements induced by cerebellar rTMS probably require neither the planning of the cerebral cortex nor the afferents of the orofacial sensation. This external stimulation may cause decreased ReHo in the cingulate gyrus, insula, temporal lobe, and supplementary motor areas. In addition, negative feedback regulation occurs between higher cortical centers and subcortical centers such as the brainstem. The elevated brainstem function after rTMS in the cerebellum may contribute to the suppression of generalized cortical function (Mosier and Bereznaya, 2001).

Our study also revealed reduced ReHo in the corpus callosum, the largest commissural connection between the cerebral hemispheres, after cerebellar rTMS. It has been suggested that the bilateral cerebral hemispheres are under interactive inhibition and that the inhibition of unilateral hand motor areas by low-frequency rTMS is beneficial by elevating contralateral hand innervation (Bajwa et al., 2008). Similarly, bilateral innervation *via* the corpus callosum may exert excitatory contralateral modulation of swallowing (Mistry et al., 2012). The findings of this study suggest that while performing bilaterally controlled swallowing movements, the inhibitory effect of the corpus callosum may be reduced.

As we all know, swallowing is divided into oral, pharyngeal, and esophageal phases. The oral phase mainly involves the formation and transport of food boluses, which are regulated autonomously by the cerebral cortex. The main activity of the pharyngeal phase is to swallow food to the esophagus, which is a reflex action, and is controlled by the CPG of the medulla oblongata (Torii et al., 2012). Our results suggest that cerebellar rTMS may not only regulate swallowing during the oral phase by inhibiting the cerebral cortex but also directly improve the performance of swallowing during the pharyngeal phase by enhancing the descending efferent pathways of the brainstem.

## Conclusion

In this study, we found that rTMS of the swallowing cortex in the dominant cerebellar hemisphere increased the bilateral cerebral swallowing cortex excitability and enhanced pontine, bulbar, and cerebellar spontaneous neural activity, suggesting that unilateral high-frequency stimulation of the cerebellum can excite both brainstem and cortical swallowing centers. Furthermore, we found reduced ReHo in the corpus callosum, which may facilitate the execution of this bilaterally innervated action of swallowing. These findings all provide favorable support for the application of cerebellar rTMS in the clinical practice.

## Limitations

Our study has some limitations. First, unlike previous clinical studies that have simulated stroke damage, we stimulated and observed healthy volunteers. This does not directly mimic the recovery process of swallowing function in stroke patients. Second, because of the higher acquisition and analysis costs of rs-fMRI, we studied a limited number of volunteers, and studies with larger samples are still needed to further confirm these conclusions.

## Data Availability Statement

The raw data supporting the conclusions of this article will be made available by the authors, without undue reservation.

## Ethics Statement

The studies involving human participants were reviewed and approved by the Ethics Committee of the Affiliated Hospital of Qingdao University. The patients/participants provided their written informed consent to participate in this study.

## Author Contributions

LD, PM, and QW contributed to conception and design of the study. WM and conducted data collection. YW and XP performed the statistical analysis. LD wrote the first draft of the manuscript. PM, QW, CH, and LD wrote sections of the manuscript. All authors contributed to manuscript revision, read, and approved the submitted version.

## Funding

This work was supported by the Key Research and Development Projects of Shandong Province (grant no. 2019GSF108262).

## Conflict of Interest

The authors declare that the research was conducted in the absence of any commercial or financial relationships that could be construed as a potential conflict of interest.

## Publisher's Note

All claims expressed in this article are solely those of the authors and do not necessarily represent those of their affiliated organizations, or those of the publisher, the editors and the reviewers. Any product that may be evaluated in this article, or claim that may be made by its manufacturer, is not guaranteed or endorsed by the publisher.
